# Loss of Reduction after Cephalomedullary Nail Fixation of Intertrochanteric Femoral Fracture: A Brief Report

**DOI:** 10.1111/os.12828

**Published:** 2020-10-20

**Authors:** Yao Pang, Qi‐fang He, Liu‐long Zhu, Zhen‐yu Bian, Mao‐qiang Li

**Affiliations:** ^1^ Department of Orthopaedic Surgery Hangzhou Hospital Affiliated to Nanjing Medical University (Hangzhou First People's Hospital) Nanjing China; ^2^ Department of Orthopaedic Surgery Hangzhou First People's Hospital Hangzhou China

**Keywords:** Cephalomedullary nail, Internal fixation, Intertrochanteric femoral fracture

## Abstract

**Objective:**

At present, cephalomedullary nail is the most frequently used implant in the management of intertrochanteric fractures around the world. The implant design and fixation techniques of the cephalomedullary nail have been continuously improved to ensure uncomplicated bone union during the past decade. However, a degree of reduction loss during bone healing is still not rare in clinical work. Many attributed this complication to misoperation during the surgery and hold that a series of techniques and tips could help to avoid the loss of reduction. However, until now there has been no research to explore whether the reduction loss after the operation can be fully prevented in the best cases. The purposes of the study are as follows: (i) to evaluate the efficiency of the current established CMN techniques; (ii) to quantify the loss of reduction under an appropriately implanted CMN to anatomically realigned intertrochanteric fractures; and (iii) to explore the possible underlying causes for the inevitable loss of reduction.

**Methods:**

In the retrospective study, 163 consecutive cases with the intertrochanteric fractures fixed with standard cephalomedullary nail technique were reviewed. The anatomical reduction and optimal positioning of the nail were confirmed by postoperative imaging. The fracture types ranged from 31‐A1.1–2.3 according to the OTA/AO fracture classification. One hundred and fifteen cases with stable fracture types (31A1.1–2.1) were allocated to Group A, and 48 cases with unstable 31A2.2–2.3 fracture types were allocated to Group B. The radiological measurements included femoral neck shortening, loss of the neck‐shaft angle, cutout, and cut‐through of the blade. The outcomes between postoperative and 1 year after the operation were evaluated and compared.

**Results:**

The patients consisted of 66 males and 97 females with an average age of 69.4 (range: 46–78, SD: 14.6) years. At the 1‐year follow‐up, no fixation failure or nonunion was observed in each group. The mean femoral neck shortening and loss of the neck‐shaft angle were 4.47 mm (range: 0.43–17.68, SD: 3.71) and 5.4° (range: 0.51–19.10, SD: 3.58) separately. The mean cutout and cut‐through were 1.84 mm (range: 0.24–11.30, SD: 2.33) and 1.25 mm (range: 0.51–10.29, SD: 1.74). The average femoral neck shortening and loss of the neck‐shaft angle were higher in Group B than Group A. Among the 23 cases with the femoral neck shortening more than 10 mm, 19 cases (16.5%) were from Group A and four cases (8.3%) were from Group B. There were nine (7.8%) cases with the loss of the neck‐shaft angle more than 10° in Group A and six (12.5%) cases in Group B.

**Conclusions:**

Current established CMN techniques are efficient in treating intertrochanteric femoral fracture. However, even with currently consensual techniques of cephalomedullary nail, the process of fracture healing still risks the loss of reduction, although the migration of the blade could be minimized. This situation may associate with the intrinsic design of the CMN and further improvement is still needed.

## Graphical abstract

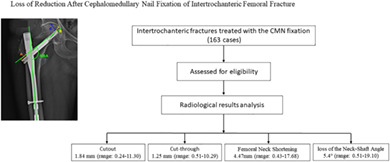

## Introduction

Hip fractures are prevelant in the elderly population and are commonly caused by a low‐energy injury mechanism such as a fall from standing height. These fractures have been recognized as the most serious consequence of osteoporosis because of its complications, which include disability, chronic pain, diminished quality of life, and premature death. With the rising life expectancy of the global population, the number of elderly individuals is increasing in every geographical region. According to the epidemiologic projections, the worldwide annual number of hip fractures is estimated to rise from 1.66 mn in 1990 to 6.26 mn by the year 2050^1^. These fractures are responsible for the largest use of resources for orthopaedics trauma around the world^2^. Approximately half of all hip fractures are intertrochanteric femoral fractures, defined as extracapsular fractures that occur between the greater and lesser trochanter of the proximal femur^3^. In patients over 50 years of age, more than 90% of hip fractures are intertrochanteric fractures with 20%–30% of these cases experiencing complications and a mortality rate of approximately 17%^4^.

As there is high morbidity and mortality associated with historical nonoperative treatment, surgical management with internal fixation is commonly necessary for these fractures. The goal of care is to restore limb function with the lowest possible rate of surgical and medical complications. Achieving stable reduction and rigid fixation of the fracture and permitting immediate mobilization are the keys to this end^5^. Dynamic hip screw (DHS) as an extramedullary construct or intramedullary nail with a cephalomedullary screw is the standard surgical treatment option chosen by most surgeons. There is literature about comparisons between these implants, including biomechanical characteristics, indications, complications, and outcomes^4^, ^6^, ^7^, ^8^, ^9^. The recent consensus is that the implant options for the treatment of intertrochanteric fractures are closely related to the stability of the fractures^10^, ^11^. According to the AO/OTA Classification of Fractures and Dislocations, there is currently little evidence of the superiority of one device over another when the intertrochanteric fracture is stable (type A1 to A2.1)^12^, ^13^. DHS has been shown to be the most cost‐effective option and has produced consistently good results in treating stable fractures. However, there are consistent concerns about the high failure rate associated with the use of the DHS in an unstable situation^14^, ^15^. A report from Evidence‐Based Working Group in Trauma analyzed all the evidence and concluded that failure rates of treatment of unstable trochanteric fractures with a DHS are too high to recommend its use^16^. In the past decade, cephalomedullary nail (CMN) has gradually become the most frequently used implant to intertrochanteric fractures due to its biomechanical advantages^17^. Studies about implant loading confirm that the load to an implant is increased with varus mal‐reduction or with decreasing stability of the fracture, and that in these cases the intramedullary device can bear greater load than the extramedullary device^18^, ^19^. Thus, current guidelines and consensus support that, in managing unstable intertrochanteric fractures, CMN is preferable to the extramedullary devices^20^.

With the increasing use of the CMN, associated failures have been continuously reported in the past decades, which typically presented as nonunion or malunion of the fractures with lag screw/blade cutout or cut‐through^21^, ^22^, ^23^. It is reported that the incidence of postoperative varus collapse with the cutout was between 1.5% and 6.5%^24^, ^25^. Besides, other complications including femoral neck shortening (FNS) and loss of neck‐shaft angle (NSA), although less emphasized by the literature, are virtually more common^26^, ^27^. Failure of fixation, nonunion, and severe malunion may lead to further revision procedures, and malunited proximal femur would causes pain and weakness of the hip although not often accompanied by significant limb disability^28^. The reasons for these failed treatments could be compounding and complicated. Several pre‐existing risk factors of fixation failure are described as the fracture type (i.e. classification), patient age, patient body weight, or bone quality^29^. The other factors, however, may directly associate with the operation procedure. Commonly accepted operative predictors for fixation failure are the quality of reduction, the tip apex distance (TAD), and lag screw position within the femoral head and neck^30^.

Nowadays, the techniques of implanting the CMN have been renewed, emphasizing on effective fracture reduction and control of the implant position^10^, ^31^, ^32^. This concept has been accepted by most orthopaedic surgeons for clinical use. However, failed cases are still reported from time to time. Thus far, it is still unconfirmed that, with optimized reduction and fixation to the fractures, whether the loss of reduction can be fully prevented, and further, whether there are still other factors in the failure mechanism. The purposes of the study are as follows: (i) to evaluate the efficiency of the current established CMN techniques; (ii) to quantify the loss of reduction under an appropriately implanted CMN to anatomically realigned intertrochanteric fractures; and (iii) to explore the possible underlying causes for the inevitable loss of reduction.

## Methods

### *Inclusion and Exclusion Criteria*

Patients meeting the following criteria were included: (i) patient diagnosed as type 31‐A1.1–2.3 intertrochanteric fracture; (ii) treated operatively and fixed with the CMN; (iii) postoperative follow‐up not less than 1 year; (iv) full postoperative radiological outcomes were acquired; (v) a retrospective study. Patients were excluded based on the following criteria: (i) pathological fractures; (ii) prior open fractures with neurovascular injury; (iii) unacceptable realignment of the fracture; (iv) malposition of the implants where the value of tip‐apex distance (TAD) is more than 25 mm, or the helical blade not placed in the center of the femoral head on anteroposterior (AP) or lateral view; (v) severe osteoporosis; (vi) patients without the ability to walk independently before the fracture.

### *Patients*

The retrospective study was approved by the institutional review board (IRB). From January 2014 to January 2016, consecutive patients with intertrochanteric fractures treated with the CMN in a Level I trauma center were reviewed.

### *Surgical Technique*

The CMN with appropriate length and diameter (The Fi‐nail, Sanatmetal Ltd., Hungary) were used in the present study. The fracture was reduced and fixed following a standard process by the same surgical team. The reduction criteria were defined as a neck‐shaft angle (NSA) over 125° and less than 20° angulation on the lateral view, as well as neutral or positive medial support^33^. During the fixation, the helical blade was placed in the center of the femoral head on anteroposterior (AP) or lateral view, and a value of tip‐apex distance (TAD) of less than 25 mm was required.

### *Postoperative Management*

Low‐molecular‐weight heparin was administered to all patients once daily for 4 weeks. Postoperative active functional exercises were encouraged. Pain‐tolerated weight‐bearing was started 4 weeks postoperatively. Full weight‐bearing was not allowed until bone union, usually 3–5 months.

### *Radiological Assessment*

**Fig. 1:**
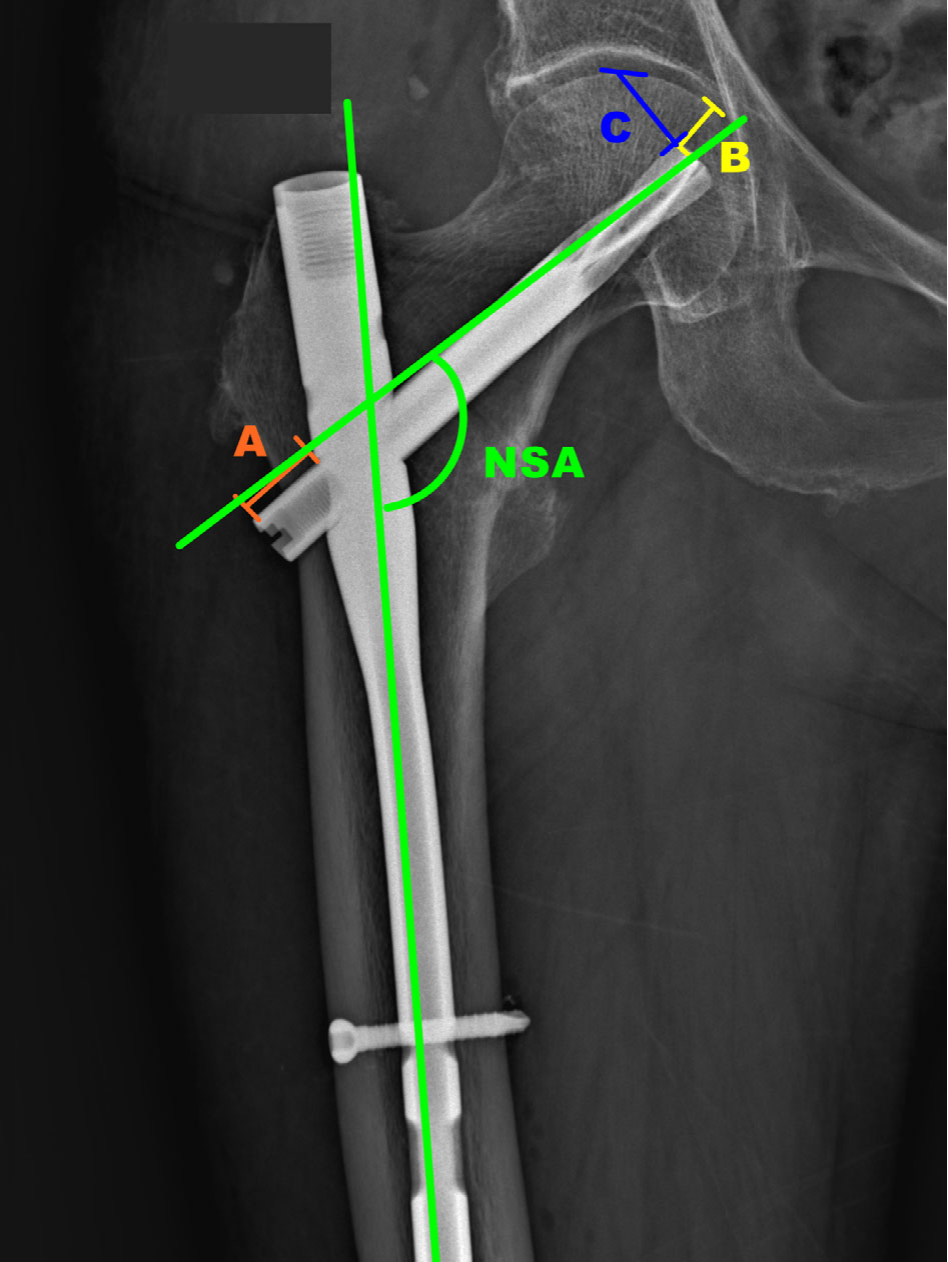
The NSA is measured as the angle between the axis of the femoral shaft and the femoral neck (green lines). The length between the distal end of the helical blade and lateral side of the nail is defined in Line A. The difference of the length of Line A between immediate postoperative and 1‐year after stands for the backout of the helical blade. The cut‐through is measured from the upper tip of the blade to the intersection of the extended line of the upper margin of the blade with the medial cortex of the femoral head (Line B). The FNS is calculated as the value of the backout plus the value of the cut‐through. Line C connects the upper tip of the blade to the intersection of the perpendicular line of the upper margin of the blade with the upper cortex of the femoral head. The cutout is calculated as the difference in the length of Line C at immediate postoperative and 1‐year after.

The radiology of all patients was obtained from Picture Archiving and Communication Systems (PACS). The radiological outcomes of immediate postoperative and 1‐year after were compared. The measurement and calculation methods were illustrated in Fig. 1.

#### *The Loss of the NSA*

The femoral NSA was defined as the angle formed by the femoral shaft with the femoral neck, which is generally set at 125°. In the study, the NSA is measured as the angle between green lines, which stand for axes of femoral shaft and femoral neck (Fig. 1). The loss of the NSA after operation indicates medial fragments collapse and varus deformity formation during the bone healing.

#### *The Cutout and Cut‐through*

The cutout was defined as “the collapse of the neck‐shaft angle into varus, leading to extrusion of the screw/blade from the femoral head,” which is the most common mechanical failure in the internal fixation of trochanteric hip fractures. The cut‐through was defined as a central perforation of the screw/blade into the hip joint without any displacement of the neck–head fragment. The distance between the upper tip of the blade to the intersection of the extended line of the upper margin of the blade with the medial cortex of the femoral head is defined as Line B. This line connects the upper tip of the blade to the intersection of the perpendicular line of the upper margin of the blade with the upper cortex of the femoral head, defined as Line C. The value of cut‐through and cutout are separately calculated as the difference of the length of Line B and Line C between immediate postoperative and 1‐year after. The value of cutout and cut‐through indicate the degree of migration of the screw/blade within the femoral head.

#### *The FNS*

The FNS in this study is defined as the difference of femoral neck length between postoperative immediate and after 1‐year measurements. The length between the distal end of the helical blade and lateral side of the nail is defined in Line A. The difference of the length of Line A between immediate postoperative and 1‐year after stands for the backout of the helical blade. The FNS is indirectly calculated as the value of the backout plus the value of the cut‐through. Shortened femoral neck would decrease the moment arm of the abductors, and increase requisite muscular force and joint reaction forces during functional activities. Excessive shortening often associates with the collapse of the fracture and malunion.

### *Statistical Analysis*

All the calculations and comparisons were achieved using IBM® SPSS® Statistics version 25 (IBM SPSS Statistics for Windows 64bit, Version 22.0; IBM® Corp., Armonk, NY, USA). The estimates from normally distributed data were reported as means, SDs, and ranges.

## Results

One hundred and sixty‐three patients consisted of 66 males and 97 females, with an average age of 69.4 (range: 46–78, SD: 14.6) years. One hundred and fifteen cases with stable fracture types (31A1.1–2.1) were classified into Group A, and 48 cases with unstable 31A2.2–2.3 fracture types were classified into Group B.

### *The Loss of the NSA*

At the 1‐year follow‐up, no fixation failure or non‐union was observed in each group. For all cases, the mean loss of the NSA was 5.4° (range: 0.51–19.10, SD: 3.58).

### *The Cutout, Cut‐through, and FNS*

The mean cutout, cut‐through, and FNS were 1.84 mm (range: 0.24–11.30, SD: 2.33), 1.25 mm (range: 0.51–10.29, SD: 1.74), and 4.47 (range: 0.43–17.68, SD: 3.71) separately. By comparison, there were more Loss of the NSA and FNS in Group B than Group A (Table 1). There were 15 cases (9.2%) with the loss of the NSA more than 10° and 23 (14.11%) cases with the FNS more than 10 mm, and 63 (38.65%) cases with the FNS more than 5 mm. Detailly, there were nine (7.8%) cases with loss of the NSA more than 10° in Group A and six (12.5%) cases in Group B. Fourteen cases (12.17%) with the FNS more than 10 mm were from Group A and nine cases (18.75%) were from Group B.

**TABLE 1:** The postoperative radiological outcomes (mean±SD)

Measurements	Group A	Group B
Loss of the NSA*	5.51 ± 0.52	8.39 ± 0.33
Cutout	1.53 ± 0.32	1.96 ± 0.23
Cut‐through	1.18 ± 0.18	1.29 ± 0.20
FNS*	3.59 ± 0.35	4.74 ± 0.50

* *P < 0.05*.

## Discussion

The systemic classifications of intertrochanteric fractures majorly include AO/OTA classification, Kyle's classification, Boyd and Griffin classification, Evans classification, and Jensen's modification of the Evans classification. Besides the description of fracture morphology, the stability associated with the fracture patterns was also commonly emphasized by almost all classifications. There is evidence that the AO/OTA classification is more reliable for the classification of intertrochanteric fractures of the proximal femur into fracture types and groups than other classification systems (e.g. Evans/Jensen and Boyd)^34^. And to experienced surgeons, AO/OTA classification shows higher agreement in the assessment of stability than others^35^. In light of that, we adopted the AO/OTA classification as the criteria of grouping in the study.

Radiologically, the typical cutout presented as penetration of the lag screw/blade into the anterior–superior femoral head accompanied with varus collapses of the fracture, which is considered as the most frequent mode of failure after CMN fixation of intertrochanteric femur fractures^36^, ^37^, ^38^. Possible causes of the failure consist of the pattern of fracture, the quality of the reduction, and the positioning of the lag screw/blade, as well as the bone quality^39^, ^40^. As the only two factors that can be controlled by the surgeon, it is well recognized that an anatomical reduction and optimal lag screw/blade position are essential to maximizing the outcome^38^. It is reported that an anatomic or slightly over‐reduced NSA prevented fracture displacement after fixation, while a realigned NSA less than the native value could associate with more varus malunion^41^. Simultaneously, a neutral or positive medial cortical support would also decrease the loss of the NSA and FNS more than those with negative medial cortical support^42^. On the basis of an appropriate reduction, the positioning of the lag screw/blade is the critical procedure during the implantation of the CMN. Generally, the center of the lag screw/blade should be located in the second quarter of the head–neck interface line (safe zone) rather than the center of the neck^43^. Moreover, a TAD over 25 mm should be avoided as it has been shown to increase the risk of cutout^44^.

**Fig. 2:**
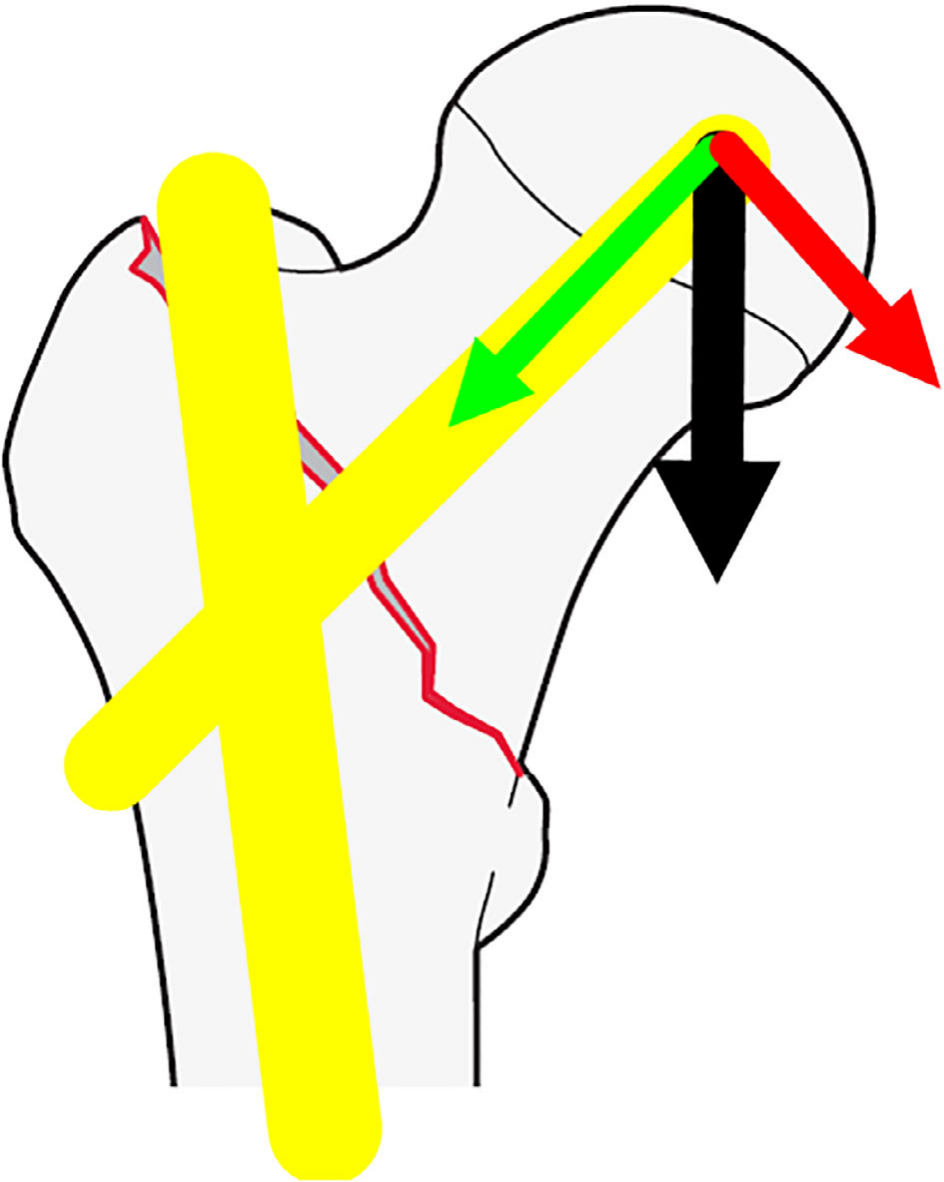
The physiological load (black arrow) bifurcates into an axial branch along the femoral neck (green arrow) and an adverse load vertical to the femoral neck (red arrow).

During the fixation, the CMN independently provides centralized support to the proximal fragment and the interfragmentary compression, namely dynamization. Through the intrinsic sliding mechanism and the “γ” shape design, the physiological vertical load was converted to an axial load compressing the fracture gap, which is regarded as beneficial to the fracture healing^45^. This axial load along the femoral neck would be accompanied by a degree of FNS. A stable intertrochanteric fracture may withstand the axial loading, but unstable fractures due to comminution are frequently associated with moderate or severe FNS under the compression^10^. According to an investigation, the FNS more than 5 mm and 10 mm occurred in 58.5% and 17% patients with OTA/AO 31‐A intertrochanteric fractures, respectively^26^. In the study, there were 63 (38.65%) cases with the FNS more than 5 mm, and 23 (14.11%) cases with the FNS more than 10 mm, and a higher incidence was associated with the unstable fractures (Group B). On the other side, the physiological load bifurcates into not only an axial branch along the femoral neck but also an adverse load vertical to the femoral neck (Fig. 2). This adverse load constantly tends to decrease the NSA or increase the cutout until fracture unites. The loss of the NSA was widely observed in both groups, and also was more severe in Group B.

Generally, the fixation failure of intertrochanteric fractures was commonly blamed on the misused techniques^46^, ^47^. However, a considerable loss of reduction was still observed with an appropriate reduction and fixation on either stable or unstable fractures. To our understanding, this result may imply that a degree of loss of reduction during the fixation is resistant, which could possibly involve the intrinsic design and compression mechanism of the CMN. The concept of limiting the sliding‐compression process has been proposed for several years, and the biomechanical and clinical outcomes are encouraging^48^. However, it remains unsettling whether an angle‐stability nail without dynamic compression would raise the risk of cutout and nonunion. A revolutionary improvement of the nail giving consideration to both stability and bone healing is in need.

## Conclusion

Current established CMN techniques are efficient in treating intertrochanteric femoral fracture. However, although implants and techniques have been optimized for the treatment of intertrochanteric fractures, the maintenance of the reduction remains defective. This situation may be associated with the intrinsic design of the CMN. Further improvement of the nails is still needed.

## Disclosure

The author declares no competing interest.
